# Long noncoding RNA HEIH promotes melanoma cell proliferation, migration and invasion via inhibition of *miR-200b/a/429*

**DOI:** 10.1042/BSR20170682

**Published:** 2017-06-21

**Authors:** Haiying Zhao, Guoping Xing, Yingying Wang, Zengxiang Luo, Guoyan Liu, Huijuan Meng

**Affiliations:** 1 Department of Dermatology, Binzhou Central Hospital, Binzhou, Shandong 251700, China; 2 Department of Neurology, Weifang People’s Hospital, Weifang, Shandong 261041, China; 3 Department of Oncology, Binzhou Central Hospital, Binzhou, Shandong 251700, China; 4 Department of Dermatology, Affiliated Hospital of Weifang Medical University, Weifang, Shandong 261031, China

**Keywords:** cell proliferation, cell migration, cell invasion, long noncoding RNA, melanoma, miR-200

## Abstract

Long noncoding RNAs (lncRNAs) are frequently dysregulated and have important roles in many diseases, particularly cancers. lncRNA-HEIH was first identified in hepatocellular carcinoma (HCC). The expression, clinical significance and roles of lncRNA-HEIH in melanoma are still unknown. In the present study, we found that lncRNA-HEIH is highly expressed in melanoma tissues and cell lines, associated with advanced clinical stages, and predicts poor outcomes in melanoma patients. Functional assays showed that ectopic expression of lncRNA-HEIH promotes melanoma cell proliferation, migration and invasion. Knockdown of lncRNA-HEIH inhibits melanoma cell proliferation, migration and invasion. Mechanistically, we revealed that lncRNA-HEIH directly binds to *miR-200b/a/429* promoter and represses *miR-200b/a/429* transcription. The expression of *miR-200b* is inversely associated with lncRNA-HEIH in melanoma tissues. Furthermore, overexpression of *miR-200b/a/429* abrogates melanoma cell proliferation, migration and invasion enhanced by lncRNA-HEIH. In conclusion, we identified *lncRNA-HEIH* as a key oncogene in melanoma via transcriptional inhibition of *miR-200b/a/429*. Our data suggested that lncRNA-HEIH may serve as a promising prognostic biomarker and therapeutic target for melanoma.

## Introduction

Melanoma, derived from pigment cells, is the most malignant skin cancer and accounts for the majority of skin cancer related deaths worldwide [1,2]. Currently, the estimated new cases of melanoma are 160000, and the estimated deaths caused by melanoma are 48000 each year [1,3]. Unfortunately, the incidence and mortality of melanoma has increased rapidly during the recent years and will continue to increase [4,5]. Despite that primary melanoma could be cured by surgical resection, melanoma is highly likely to metastasize, and the metastatic melanoma has high mortality and poor prognosis [6]. Therefore, uncovering the molecular mechanisms driving melanoma tumorigenesis and progression, identifying biomarkers for early diagnosis of melanoma and developing effective melanoma therapeutic strategies are urgently needed [7].

With the development of high-throughput RNA sequencing technology, approximately 70–90% of human genome is revealed to be transcribed into RNA, but over 68% of transcripts are classified as noncoding RNAs [8,9]. Formerly, the few observed noncoding RNAs were regarded as dark matters and transcribed noise of the genome. However, increasing evidence revealed that noncoding RNAs play important roles in various pathophysiological processes and are frequently dysregulated in many diseases [10–15]. The huge number and important functions of noncoding RNAs prompt us to re-evaluate and further explore these noncoding RNAs in human diseases.

According to the length, noncoding RNAs are classified into long noncoding RNAs (lncRNAs) (>200 nts) and small noncoding RNAs (<200 nts) [16–20]. To date, only a few lncRNAs have been studied in melanomas. lncRNAs SAMMSON, MHENCR, SLNCR1, PVT1 and MALAT1 are increased and have pro-oncogenic functions in melanoma [21–25]. lncRNA NKILA is decreased and have tumour suppressing functions in melanoma [26]. The expressions and functions of other lncRNAs need further investigation in melanoma. lncRNA-HEIH was first reported to be up-regulated in hepatocellular carcinoma (HCC), indicating poor outcome of HCC patients and promotes cell-cycle progression of HCC cells [27]. However, the expression, clinical significances and biological roles of lncRNA-HEIH in melanoma are still unknown.

In the present study, we investigated the expression of lncRNA-HEIH in melanoma, explored the correlation between lncRNA-HEIH expression and clinicopathological features and prognosis of melanoma patients, assessed the roles of lncRNA-HEIH in melanoma cell proliferation, migration and invasion, and studied the molecular mechanisms underlying the roles of lncRNA-HEIH in melanoma.

## Materials and methods

### Clinical tissue samples

The Ethics Committee of the Affiliated Hospital of Weifang Medical University reviewed and approved the use of clinical tissues samples. Sixty-six melanoma issues and 42 benign nevi were obtained from patients who underwent surgical resection at the Affiliated Hospital of Weifang Medical University. None of the patients received preoperative adjuvant treatment. The tissue specimens were diagnosed by pathological examination. All the patients signed written informed consents prior to the study.

### Cell culture

The human epidermal melanocyte HEMa-LP was purchased from Invitrogen (Carlsbad, CA, U.S.A.). The melanoma cell lines SK-MEL-28, A375, A2058 and SK-MEL-2 were obtained from American Type Culture Collection (ATCC). HEMa-LP was cultured in Medium 254 and Human Melanocyte Growth Supplement-2 (Invitrogen). SK-MEL-28 and SK-MEL-2 were cultured in Eagle’s minimum essential medium (Invitrogen). A375 and A2058 were cultured in Dulbecco’s modified Eagle’s medium (Invitrogen). All the cells were cultured in medium containing 10% FBS (Gibco BRL, Gaithersburg, MD, U.S.A.) at 37°C with 5% CO_2_ and saturated humidity.

### RNA extraction and quantitative real-time PCR

TRIzol reagent (Invitrogen) was used to extract RNA from tissues and cells in accordance with the manufacturer’s instructions. After the removal of genomic DNA using DNase I, reverse transcription was carried out using equal amounts of RNA and M-MLV Reverse Transcriptase (Invitrogen) to generate the first-strand cDNA. Quantitative real-time PCR (qRT-PCR) was carried out using SYBR® Premix Ex Taq™ II (TaKaRa Biotechnology Ltd., Dalian, China) on StepOne Plus Real-Time PCR System (Applied Biosystems, Foster City, CA, U.S.A.) in accordance with the manufacturer’s instructions. β-actin was used as an endogenous control for lncRNAs. The primers sequences are as follows: for lncRNA-HEIH: 5′-CTCTTGTGCCCCTTTCTT-3′ (sense) and 5′-ATGGCTTCTCGCATCCTAT-3′ (antisense); for β-actin, 5′-GGGAAATCGTGCGTGACATTAAG-3′ (sense) and 5′-TGTGTTGGCGTACAGGTCTTTG-3′ (antisense). For miRNAs detection, qRT-PCR was carried out using TaqMan miRNA assays (Applied Biosystems) in accordance with the manufacturer’s instructions. U6 was used as an endogenous control for miRNAs. The comparative *C*_t_ method was performed to calculate the expression of target genes.

### Vectors construction and transfection

Full-length lncRNA-HEIH was PCR amplified with the Phusion Flash High-Fidelity PCR Master Mix (Thermo Fisher, Waltham, MA, U.S.A.) and inserted into the *Kpn*I and *Xba*I sites of pcDNA3.1 (Invitrogen), termed pcDNA3.1-HEIH. The primers sequences are as follows: 5′-GGGTACCGTCCCCGCCCCCTGCTG-3′ (forward) and 5′-GCTCTAGACAAGGTTGGAAAATCCCACTTTAC-3′ (reverse). Two independent shRNAs specifically targeting lncRNA-HEIH were designed and synthesized by GenePharma (Shanghai, China), termed as shRNA-HEIH-1 and shRNA-HEIH-2. The shRNA sequences are as follows: for shRNA-HEIH-1: 5′-TGCGCCTTCCCTCTAACCTTAATTCAAGAGATTAAGGTTAGAGGGAAGGCGCTTTTTTC-3′; for shRNA-HEIH-2: 5′-TGGCAAGATGAACGTCTGAAATTTCAAGAGAATTTCAGACGTTCATCTTGCCTTTTTTC-3′. A scrambled shRNA was used as a negative control (NC) for shRNA-HEIH-1 and shRNA-HEIH-2, termed as shRNA-NC.

The double-stranded miRNAs mimics and NC (miR-NC) were purchased from GenePharma. The vectors and miRNAs were transfected into melanoma cells using Lipofectamine 3000 (Invitrogen) in accordance with the manufacturer’s instructions.

### Establishment of lncRNA-HEIH stably overexpressed and knocked down melanoma cells

To obtain lncRNA-HEIH stably overexpressed and control A375 cells, pcDNA3.1-HEIH or pcDNA3.1 was transfected into A375 cells. Then, the cells were selected with 800 μg/ml neomycin for 4 weeks. To obtain lncRNA-HEIH stably depleted and control A2058 cells, shRNA-HEIH-1, shRNA-HEIH-2, or shRNA-NC was transfected into A2058 cells. Then the cells were selected with 800 μg/ml neomycin for 4 weeks. The overexpression and knockdown efficiencies of the stable cells were confirmed by qRT-PCR.

### Cell proliferation assays

Glo cell viability assays and ethynyl deoxyuridine (EdU) incorporation assays were carried out to assess cell proliferation potential. For Glo cell viability assays, a total of approximately 2000 melanoma cells/well were seeded in 96-well plate. After culturing for 24, 48 and 72 h, cell viability was assessed using the CellTiter-Glo® Luminescent Cell Viability Assay (Promega, Madison, WI, U.S.A.) in accordance with the manufacturer’s instructions. EdU incorporation assays were carried out with an EdU kit (Roche, Mannheim, Germany) also in accordance with the manufacturer’s instructions.

### Transwell assays

Transwell assays were carried out to assess cell migration potential. Briefly, indicated melanoma cells suspended in serum-free medium with 1 μg/ml mitomycin C were plated in the upper chamber of a 24-well transwell insert (Millipore, Bedford, MA, U.S.A.). For invasion assays, Matrigel (Gibco) was used to coat the upper chamber of transwell insert. The lower chamber was filled with medium containing 10% FBS. After incubation for 24 h, cells remaining on the upper surface of the insert were scraped off with a cotton swab, and cells on the lower surface were fixed with methanol, stained with Crystal Violet and counted using Zeiss Axiophot Microscope (Carl Zeiss, Oberkochen, Germany).

### Chromatin isolation by RNA purification

Chromatin isolation by RNA purification (ChIRP) assays were carried out using the EZ-Magna ChIRP RNA Interactome Kits (No. 17-10495, Millipore, Bedford, MA, U.S.A.) in accordance with the manufacturer’s instructions. Biotin-labelled antisense oligodeoxynucleotide probes complementary to lncRNA-HEIH were designed and synthesized by Biosearch Technologies (Petaluma, CA, U.S.A.). The probes’ sequences are as follows: 1: 5′-GAGGGAAACCTTCCGGACAC-3′; 2: 5′-ACAAAAGCAGACTAGGGCGG-3′; 3: 5′-AATACTACCTTCCAGCTGTC-3′; 4: 5′-TGAGGGCGGAATACTACCTT-3′; 5: 5′-GGTATGTGATGCGAGCACAG-3′; 6: 5′-TCTTTAAGCCATTGTCTTGT-3′; 7: 5′-GTGTACTCAGAATGGAGGGG-3′; 8: 5′-ATCCCACTTTACTTCAAGTG-3′. Retrieved DNA and RNA was quantified by qRT-PCR as described above. The primers sequences for *miR-200b/a/429* promoter are as follows: 5′-CTGCGTCACCGTCACTGG-3′ (forward) and 5′-ACAACTCGCCCGTCTCTG-3′ (reverse).

### Statistical analysis

The GraphPad Prism Software was used to analyse the statistical differences. For comparisons among groups, Mann–Whitney test, log-rank test, Student’s *t* test and Pearson correlation analysis were carried out as indicated. *P*<0.05 was considered as statistically significant.

## Results

### lncRNA-HEIH is highly expressed in melanoma and predicts poor outcome in melanoma patients

**Figure 1:**
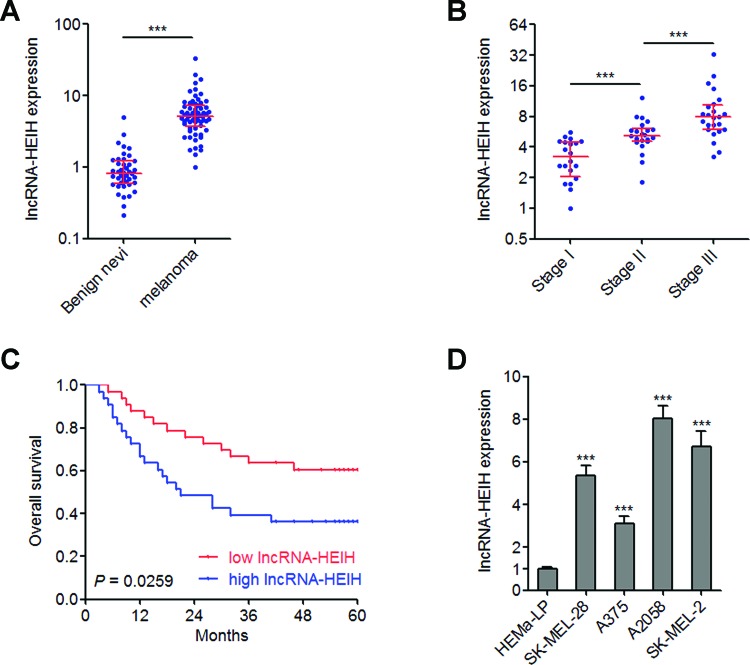
Expression of lncRNA-HEIH in melanoma and its association with melanoma patients’ outcomes (**A**) lncRNA-HEIH expression levels in 66 melanoma issues and 42 benign nevi were detected by qRT-PCR. Results are shown as median with interquartile range. ***P<0.001 by Mann–Whitney test. (**B**) lncRNA-HEIH expression levels in melanoma tissues with different clinical stages were detected by qRT-PCR. Results are shown as median with interquartile range. ***P<0.001 by Mann–Whitney test. (**C**) Kaplan–Meier survival analyses of the correlation between lncRNA-HEIH expression level and overall survival of melanoma patients. The median expression level of lncRNA-HEIH was used as the cutoff. *P*=0.0259 by log-rank test. (**D**) lncRNA-HEIH expression levels in human epidermal melanocyte HEMa-LP and melanoma cell lines SK-MEL-28, A375, A2058 and SK-MEL-2 were detected by qRT-PCR. Results are shown as mean ± S.D. from three independent experiments. ***P<0.001 by Student’s *t* test.

qRT-PCR was performed to detect the expression of lncRNA-HEIH in 66 melanoma issues and 42 benign nevi. The results showed that lncRNA-HEIH is highly expressed in melanoma tissues than in benign nevi (Figure 1A). Analyses of the association between lncRNA-HEIH expression and clinicopathological characteristics of these 66 melanoma patients showed that lncRNA-HEIH is highly expressed in melanoma tissues with advanced clinical stages than in early stages (Figure 1B). In addition, Kaplan–Meier survival analysis showed that high lncRNA-HEIH expression in melanoma tissues indicates poor overall survival (Figure 1C). The expression of lncRNA-HEIH in human epidermal melanocyte HEMa-LP and melanoma cell lines SK-MEL-28, A375, A2058 and SK-MEL-2 were measured. The results showed that lncRNA-HEIH is highly expressed in melanoma cell lines than in melanocyte (Figure 1D). Collectively, these data showed that lncRNA-HEIH is highly expressed in melanoma, correlated with advanced clinical stages and predicts poor outcome in melanoma patients.

### Ectopic expression of lncRNA-HEIH promotes melanoma cell proliferation, migration and invasion

**Figure 2:**
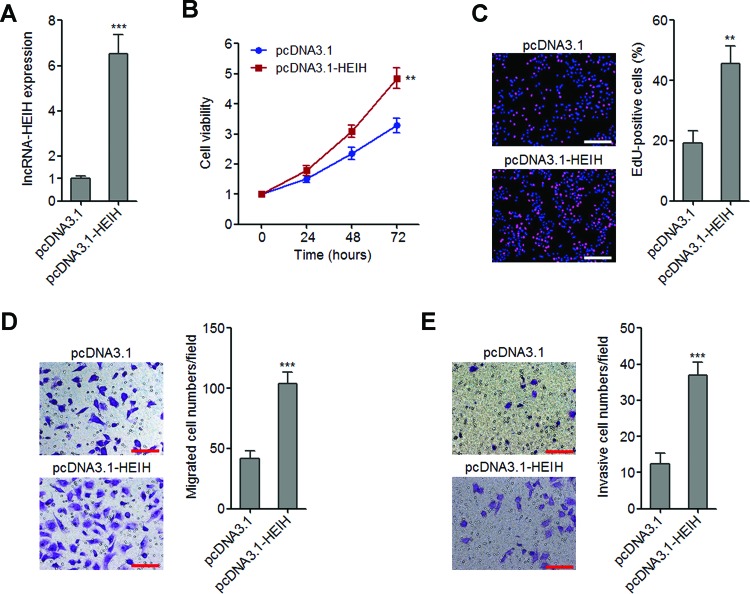
The effects of lncRNA-HEIH overexpression on melanoma cell proliferation, migration and invasion (**A**) lncRNA-HEIH expression levels in lncRNA-HEIH stably overexpressed and control A375 cells were detected by qRT-PCR. (**B**) Cell viabilities of lncRNA-HEIH stably overexpressed and control A375 cells at 0, 24, 48 and 72 h were detected by Glo cell viability assays. The data were normalized to viability at 0 h. (**C**) Cell proliferation of lncRNA-HEIH stably overexpressed and control A375 cells was detected by EdU incorporation assays. The red colour represents EdU-positive nuclei. Scale bars =200 µm. (**D**) Cell migration of lncRNA-HEIH stably overexpressed and control A375 cells was detected by transwell assays. Scale bars =100 µm. (**E**) Cell invasion of lncRNA-HEIH stably overexpressed and control A375 cells was detected by transwell assays with matrigel. Scale bars =100 µm. Results as shown as mean ± S.D. from three independent experiments. **P<0.01, ***P<0.001 by Student’s *t* test.

To explore the biological effects of lncRNA-HEIH on melanoma, we constructed lncRNA-HEIH stably overexpressed A375 cells by transfecting lncRNA-HEIH expressing plasmid pcDNA3.1-HEIH. The expression of lncRNA-HEIH was confirmed by qRT-PCR (Figure 2A). The effects of lncRNA-HEIH on A375 cell proliferation were evaluated by Glo cell viability assay and EdU incorporation assays. The growth curves determined by Glo cell viability assays revealed that ectopic expression of lncRNA-HEIH promotes A375 cell proliferation (Figure 2B). EdU incorporation assays also revealed that ectopic expression of lncRNA-HEIH increases the number of EdU-positive cells (Figure 2C). Next, the effects of lncRNA-HEIH on A375 cell migration and invasion were evaluated by transwell assays. The results demonstrated that ectopic expression of lncRNA-HEIH significantly promotes A375 cell migration and invasion (Figure 2D,E). Collectively, these data showed that ectopic expression of lncRNA-HEIH promotes melanoma cell proliferation, migration and invasion.

### Knockdown of lncRNA-HEIH inhibits melanoma cell proliferation, migration and invasion

**Figure 3:**
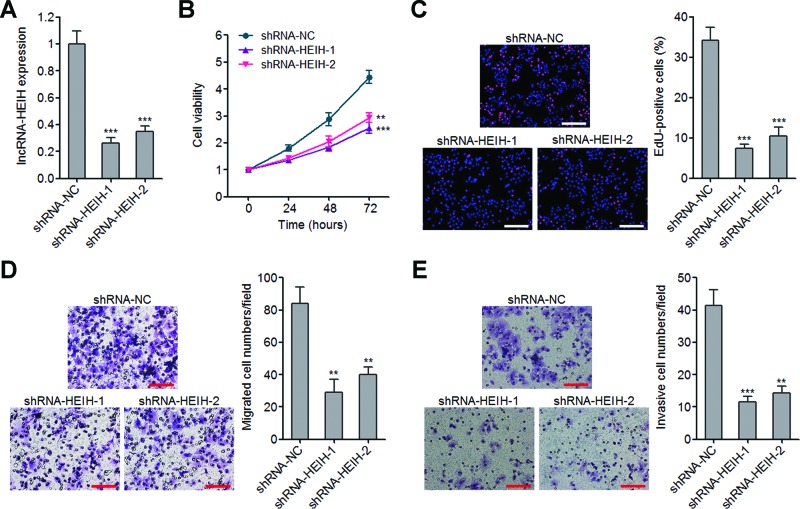
The effects of lncRNA-HEIH knockdown on melanoma cell proliferation, migration and invasion (**A**) lncRNA-HEIH expression levels in lncRNA-HEIH stably knocked down and control A2058 cells were detected by qRT-PCR. (**B**) Cell viabilities of lncRNA-HEIH stably knocked down and control A2058 cells at 0, 24, 48 and 72 h were detected by Glo cell viability assays. The data were normalized to viability at 0 h. (**C**) Cell proliferation of lncRNA-HEIH stably knocked down and control A2058 cells was detected by EdU incorporation assays. The red colour represents EdU-positive nuclei. Scale bars =200 µm. (**D**) Cell migration of lncRNA-HEIH stably knocked down and control A2058 cells was detected by transwell assays. Scale bars =100 µm. (**E**) Cell invasion of lncRNA-HEIH stably knocked down and control A2058 cells was detected by transwell assays with matrigel. Scale bars =100 µm. Results are shown as mean ± S.D. from three independent experiments. **P<0.01, ***P<0.001 by Student’s *t* test.

To further confirm the biological effects of lncRNA-HEIH on melanoma, we constructed lncRNA-HEIH stably knocked down A2058 cells by transfecting two independent lncRNA-HEIH-specific shRNAs. The knockdown efficiencies of these two lncRNA-HEIH shRNAs were confirmed by qRT-PCR (Figure 3A). The growth curves determined by Glo cell viability assays revealed that knockdown of lncRNA-HEIH inhibits A2058 cell proliferation (Figure 3B). EdU incorporation assays also revealed that knockdown of lncRNA-HEIH significantly decreases the number of EdU-positive cells (Figure 3C). Next, the effects of lncRNA-HEIH knockdown on A2058 cell migration and invasion were evaluated by transwell assays. The results demonstrated that knockdown of lncRNA-HEIH inhibits A2058 cell migration and invasion (Figure 3D,E). Collectively, these data showed that knockdown of lncRNA-HEIH inhibits melanoma cell proliferation, migration and invasion.

### lncRNA-HEIH binds to *miR-200b/a/429* promoter and inhibits *miR-200b/a/429* transcription

**Figure 4:**
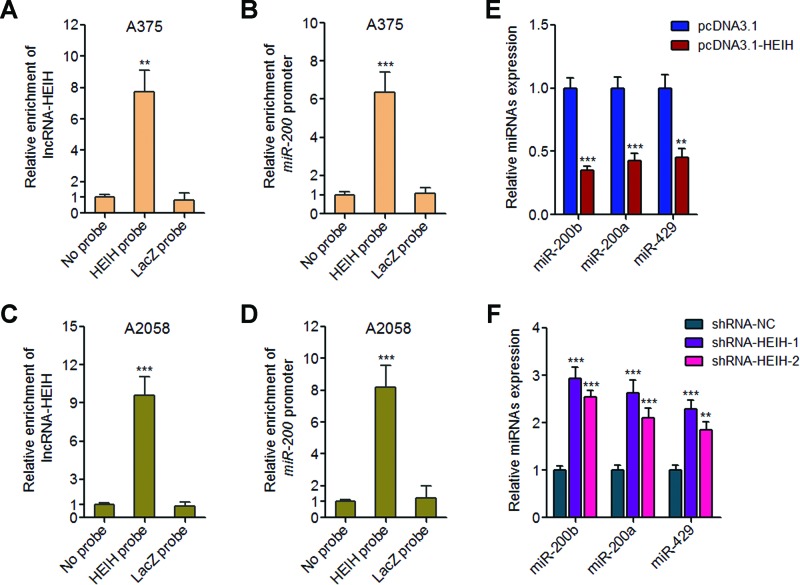
lncRNA-HEIH binds to *miR-200b/a/429* promoter and suppresses *miR-200b/a/429* expression (**A**) ChIRP assays in A375 cells were carried out with biotin-labelled antisense oligodeoxynucleotide probes complementary to lncRNA-HEIH or LacZ (NC), and the retrieved RNA was detected by qRT-PCR to measure lncRNA-HEIH. (**B**) ChIRP assays in A375 cells were carried out with biotin-labelled antisense oligodeoxynucleotide probes complementary to lncRNA-HEIH or LacZ (NC), and the retrieved DNA was detected by qPCR to measure *miR-200b/a/429* promoter. (**C**) ChIRP assays in A2058 cells were carried out with biotin-labelled antisense oligodeoxynucleotide probes complementary to lncRNA-HEIH or LacZ (NC), and the retrieved RNA was detected by qRT-PCR to measure lncRNA-HEIH. (**D**) ChIRP assays in A2058 cells were carried out with biotin-labelled antisense oligodeoxynucleotide probes complementary to lncRNA-HEIH or LacZ (NC), and the retrieved DNA was detected by qPCR to measure *miR-200b/a/429* promoter. (**E**) *miR-200b/a/429* expression levels in lncRNA-HEIH stably overexpressed and control A375 cells were detected by qRT-PCR. (**F**) *miR-200b/a/429* expression levels in lncRNA-HEIH stably knocked down and control A2058 cells were detected by qRT-PCR. Results are shown as mean ± S.D. from three independent experiments. **P<0.01, ***P<0.001 by Student’s *t* test.

lncRNA-HEIH has been reported to interact with enhancer of zeste homologue 2 (EZH2), change the genomic occupation of EZH2 on its target genes’ promoters and modulate the expression of EZH2 target genes in HCC [27]. Furthermore, the critical tumour suppressors *miR-200b/a/429* have been reported to be EZH2 target genes in cervical cancer and HCC [28,29]. To investigate whether lncRNA-HEIH regulates *miR-200b/a/429* in melanoma cells, we first detected whether lncRNA-HEIH binds to *miR-200b/a/429* promoter using ChIRP assays with biotin-labelled antisense oligodeoxynucleotide probes complementary to lncRNA-HEIH. The results showed that the probes not only pull down lncRNA-HEIH, but also *miR-200b/a/429* promoter in A375 cells (Figure 4A,B). The same results were acquired with A2058 cells (Figure 4C,D). These results suggested that lncRNA-HEIH has a significant genomic occupancy on *miR-200b/a/429* promoter. qRT-PCR results showed that ectopic expression of lncRNA-HEIH inhibits *miR-200b/a/429* expression, while knockdown of lncRNA-HEIH up-regulates *miR-200b/a/429* expression (Figure 4E,F). Collectively, these data demonstrated that lncRNA-HEIH directly binds to *miR-200b/a/429* promoter and further inhibits *miR-200b/a/429* expression.

### The expression of *miR-200b* is inversely associated with lncRNA-HEIH in melanoma tissues

**Figure 5:**
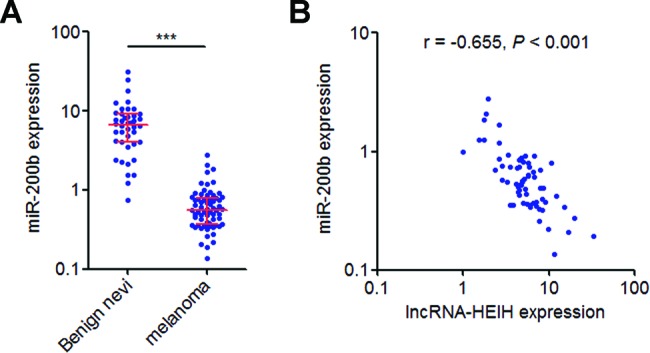
*miR-200b* expression is inversely associated with lncRNA-HEIH in melanoma tissues (**A**) *miR-200b* expression level in 66 melanoma issues and 42 benign nevi were detected by qRT-PCR. Results are shown as median with interquartile range. ***P<0.001 by Mann–Whitney test. (**B**) The correlation between lncRNA-HEIH and *miR-200b* expression levels in melanoma tissues was detected by Pearson’s correlation analysis.

To explore whether the regulation of *miR-200b/a/429* by lncRNA-HEIH also exists in clinical tissue samples, we measured the expression of *miR-200b* and analysed its correlation with lncRNA-HEIH in the same melanoma issues as shown in Figure 1A. qRT-PCR results showed that *miR-200b* is expressed lower in melanoma tissues than that in benign nevi (Figure 5A). Pearson’s correlation analyses showed that *miR-200b* expression is inversely associated with lncRNA-HEIH in melanoma tissues (*r* = –0.655, *P*<0.001) (Figure 5B), supporting the regulation of *miR-200b* by lncRNA-HEIH in melanoma tissues.

### *miR-200b/a/429* mediate the roles of lncRNA-HEIH on melanoma cell proliferation, migration and invasion

**Figure 6:**
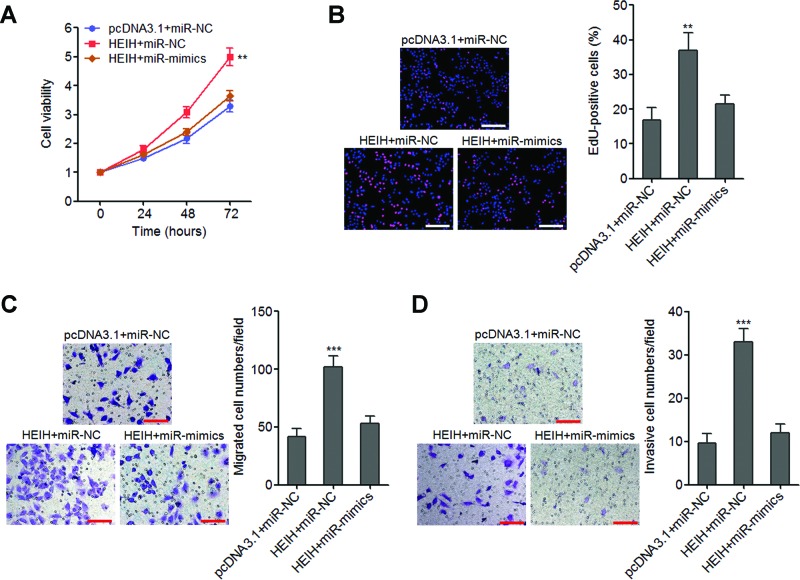
Overexpression of *miR-200b/a/429* abrogates the effects of lncRNA-HEIH on melanoma cell proliferation, migration and invasion (**A**) After transfection of the mix of *miR-200b/a/429* mimics into lncRNA-HEIH stably overexpressed A375 cells, cell viabilities at 0, 24, 48 and 72 h were detected by Glo cell viability assays. The data were normalized to viability at 0 h. (**B**) After transfection of the mix of *miR-200b/a/429* mimics into lncRNA-HEIH stably overexpressed A375 cells, cell proliferation was detected by EdU incorporation assays. The red colour represents EdU-positive nuclei. Scale bars =200 µm. (**C**) After transfection of the mix of *miR-200b/a/429* mimics into lncRNA-HEIH stably overexpressed A375 cells, cell migration was detected by transwell assays. Scale bars =100 µm. (**D**) After transfection of the mix of *miR-200b/a/429* mimics into lncRNA-HEIH stably overexpressed A375 cells, cell invasion was detected by transwell assays with matrigel. Scale bars =100 µm. Results are shown as mean ± S.D. from three independent experiments. **P<0.01, ***P<0.001 by Student’s *t* test.

To investigate whether *miR-200b/a/429* mediate the biological roles of lncRNA-HEIH on melanoma cell proliferation, migration and invasion, we transfected the mix of *miR-200b* mimics, *miR-200a* mimics and *miR-429* mimics into lncRNA-HEIH stably overexpressed A375 cells. The growth curves determined by Glo cell viability assays revealed that ectopic expression of *miR-200b/a/429* abrogates the pro-proliferative roles of lncRNA-HEIH in A375 cells (Figure 6A). EdU incorporation assays further revealed that ectopic expression of *miR-200b/a/429* abrogates the increase in EdU-positive cells number caused by lncRNA-HEIH overexpression (Figure 6B). Transwell assays revealed that ectopic expression of *miR-200b/a/429* abrogates the promigratory and proinvasive roles of lncRNA-HEIH in A375 cells (Figure 6C,D). Collectively, these data showed that the roles of lncRNA-HEIH on melanoma cell proliferation, migration and invasion are mediated by *miR-200b/a/429*.

## Discussion

With great advances in the understanding of molecular mechanisms underlying melanoma tumorigenesis and development, the immunotherapy and molecular targeted therapies have successfully extended the survival of melanoma patients [30,31]. However, most of these patients suffer recurrence and deaths due to this malignant disease [32,33] therefore a more complete understanding of mechanisms and developing novel efficient targeted therapies would greatly improve the outcomes of melanoma patients [34].

Accumulating evidence revealed that most of the human transcriptomes are noncoding RNAs [35,36]. However, the attention of melanoma was mainly focused on the protein-coding genes. Uncovering the critical roles of lncRNAs may offer new insights into and therapeutic opportunities for melanoma. lncRNA-HEIH is a recently identified lncRNA in HCC [27]. In the present study, we found that lncRNA-HEIH is up-regulated in melanoma tissues and cell lines. Increased expression of lncRNA-HEIH is associated with advanced clinical stages of melanoma. Moreover, high expression of lncRNA-HEIH predicts poor outcomes in melanoma patients. Our data suggest that lncRNA-HEIH may serve as a prognostic biomarker for melanoma. Gain-of-function and loss-of-function assays showed that ectopic expression of lncRNA-HEIH promotes melanoma cell proliferation, migration and invasion, while knockdown of lncRNA-HEIH inhibits melanoma cell proliferation, migration and invasion. These data suggest that lncRNA-HEIH has pro-oncogenic roles in melanoma.

The mechanisms underlying the roles of lncRNAs are complex [37]. lncRNAs could directly bind to proteins, mRNAs, miRNAs or DNAs, and then modulate the expression, function or localization of their target genes [38–43]. In this study using ChIRP assays, we found that lncRNA-HEIH directly binds to *miR-200b/a/429* promoter and inhibits *miR-200b/a/429* expression. *miR-200b, miR-200a* and* miR-429* belong to the *miR-200* family. The *miR-200* family has well-known tumour suppressors and modulates cell proliferation, migration, invasion, epithelial–mesenchymal transition, drug resistance etc. in many cancers, including melanoma [44–47]. In the present study, we also found that overexpression of *miR-200b/a/429* inhibits cell proliferation, migration and invasion promoted by lncRNA-HEIH. An inverse correlation between *miR-200b* expression and lncRNA-HEIH was also observed in melanoma tissues. Collectively, these data suggest that lncRNA-HEIH promotes melanoma cell proliferation, migration and invasion via inhibiting *miR-200b/a/429*. lncRNA-GIHCG is reported to repress *miR-200b/a/429* expression via physically associating with and recruiting EZH2 and DNMT1 to *miR-200b/a/429* promoter and increasing histone H3K27 trimethylation and DNA methylation levels on *miR-200b/a/429* promoter [29]. Interestingly, in HCC cells, lncRNA-HEIH was reported to be associated with EZH2 and repress EZH2 target genes, including p15, p16, p21 and p57 [27]. Whether or not the epigenetic modification enzymes, the histone and DNA mehtylation changes are reuqired for the inhibition of miR-200b/a/429 by lncRNA-HEIH need further investigation

Taken together, our data revealed that lncRNA-HEIH is highly expressed in melanoma, associated with advanced clinical stages, indicts poor prognosis in melanoma patients and promotes melanoma cell proliferation, migration and invasion. Mechanistically, lncRNA-HEIH directly binds to *miR-200b/a/429* promoter and inhibits *miR-200b/a/429* transcription. Our findings indicate that lncRNA-HEIH serve as a key regulator in melanoma and may be a promising target in melanoma treatment.

## Abbreviations

ChIRP: chromatin isolation by RNA purification
EdU: ethynyl deoxyuridine
EZH2: enhancer of zeste homologue 2
HCC: hepatocellular carcinoma
lncRNA: long noncoding RNA
NC: negative control
qRT-PCR: quantitative real-time PCR
